# Conditional Entropy and Location Error in Indoor Localization Using Probabilistic Wi-Fi Fingerprinting

**DOI:** 10.3390/s16101636

**Published:** 2016-10-02

**Authors:** Rafael Berkvens, Herbert Peremans, Maarten Weyn

**Affiliations:** 1 iMinds, MOSAIC, University of Antwerp, Faculty of Applied Engineering, Antwerp 2020, Belgium; maarten.weyn@uantwerpen.be; 2 Engineering Management, University of Antwerp, Faculty of Applied Economics, Antwerp 2000, Belgium; herbert.peremans@uantwerpen.be

**Keywords:** location error, uncertainty, conditional entropy, localization, Wi-Fi, fingerprinting, indoor, information theory

## Abstract

Localization systems are increasingly valuable, but their location estimates are only useful when the uncertainty of the estimate is known. This uncertainty is currently calculated as the location error given a ground truth, which is then used as a static measure in sometimes very different environments. In contrast, we propose the use of the conditional entropy of a posterior probability distribution as a complementary measure of uncertainty. This measure has the advantage of being dynamic, i.e., it can be calculated during localization based on individual sensor measurements, does not require a ground truth, and can be applied to discrete localization algorithms. Furthermore, for every consistent location estimation algorithm, both the location error and the conditional entropy measures must be related, i.e., a low entropy should always correspond with a small location error, while a high entropy can correspond with either a small or large location error. We validate this relationship experimentally by calculating both measures of uncertainty in three publicly available datasets using probabilistic Wi-Fi fingerprinting with eight different implementations of the sensor model. We show that the discrepancy between these measures, i.e., many location estimates having a high location error while simultaneously having a low conditional entropy, is largest for the least realistic implementations of the probabilistic sensor model. Based on the results presented in this paper, we conclude that conditional entropy, being dynamic, complementary to location error, and applicable to both continuous and discrete localization, provides an important extra means of characterizing a localization method.

## 1. Introduction

The location of the user is increasingly important information for context-aware applications. Examples are home automation [1], where heating and lighting automatically switches off when residents are away; smart cities that provide services to visitors and inhabitants through smart devices [2]; or a user at a shopping mall who wishes to know how to navigate to a particular shop [3]. Ideally, apart from the location estimate itself, the uncertainty of this location estimate is also provided, so that applications can choose between obtaining a higher certainty or using the current location estimate, thus improving other factors, such as interaction speed or energy efficiency.

The uncertainty of this location estimate is often expressed as the expected location error, that is the difference between the location estimate and the actual location [4]. However, this approach requires ground truth data. Consequently, it cannot be dynamically calculated taking into account the actual measurements from the localization sensors. Moreover, a static measure such as the median location error is given to express the uncertainty over the location estimate; there is no way of knowing if the current result belongs to the fifty percent that is better than this median error or to the fifty percent that is worse. Alternatively, one could choose to set the expected error to a certain confidence interval, for example the 95th percentile. The chance of the error being larger than the reported error would then only be 5 %. However, in that case, the system would often be underestimating its accuracy.

Therefore, a dynamic indication of the uncertainty of the location estimate based on the current sensor measurement would be more useful. In this paper, we propose to derive such a measure from the evaluation of the posterior probability distribution over the locations in an environment without the explicit need for a ground truth and use it in a Wi-Fi fingerprinting localization system. In our previous work [5], we showed a correlation between the mutual information rate and the quality of the pose graph generated by the biologically-inspired Simultaneous Localization And Mapping (SLAM) system called RatSLAM [6]. Such an evaluation using metrics from information theory was also performed on the sonar-based RatSLAM system called BatSLAM [7]. From these works, which use information theory to quantify the uncertainty in the posterior probability distribution over the locations in an environment, we propose that there is a relation between the conditional entropy of the posterior probability distribution and the location error, as they both measure location uncertainty. However, the conditional entropy has the important benefit that it can be calculated for any measurement without requiring a ground truth.

We further propose to use the relation between these two measures of uncertainty to evaluate the quality of the sensor models required for probabilistic localization. We consider a high quality sensor model to be one that models the uncertainty present in the sensor measurements correctly. Using such a sensor model, the resulting uncertainty of the location estimate (after taking into account the measurement) would also be accurately represented by the posterior probability distribution of this estimate. Using conditional entropy as a measure of the uncertainty of the location estimate remaining after the measurement, we will show that low quality sensor models, i.e., ones that insufficiently take into account the sources of uncertainty present in the sensor measurements, result in location estimates that are simultaneously highly certain and far removed from the true location. While the occurrence of location estimates that have both low conditional entropy and large errors cannot be used to derive the changes that need to be made to the sensor model, we propose it is nevertheless quite useful as a way of detecting an insufficiently accurate sensor model. We illustrate the use of this measure for the purpose of improving the sensor model, i.e., improving its modeling of the various sources of uncertainty, by the analyses presented in the paper. We are strongly convinced that improving a sensor model in this sense, while not necessarily leading to more accurate location estimates, could nevertheless improve the overall performance of many localization applications; in particular, those applications where knowledge of the uncertainty of a location estimate is as important as the location estimate itself.

We will use the relation between conditional entropy and location error to analyze the performance of probabilistic sensor models used in Wi-Fi fingerprinting, thereby improving and extending our preliminary results [8]. Our choice of Wi-Fi fingerprinting allows us to use publicly-available datasets. The three datasets are: a dataset from the EVARILOS [9] European project, in the w-iLab.t II environment; GEOTEC [10], a smaller database in an office environment; and UJIIndoorLoc [11], a large dataset created in three buildings of up to four floors.

**Figure 1:**
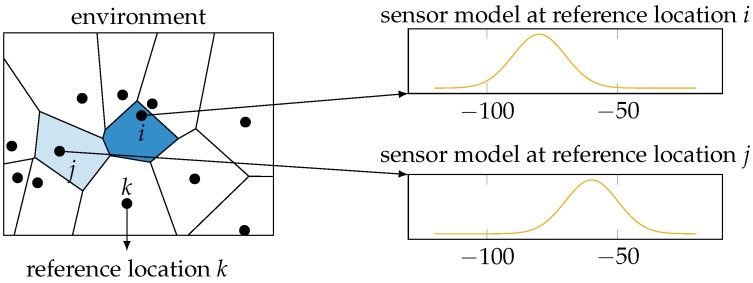
The environment in Wi-Fi fingerprinting is split up into discrete spaces, namely the nearest locations to a single reference location. The sensor model’s dependency on the location parameters changes discontinuously as it is determined by the training measurements at the reference locations.

Note that our choice of Wi-Fi fingerprinting excludes the use of more widely-used dynamic metrics to measure location uncertainty, most notably the Cramér-Rao lower bound (CRLB) and the dilution of precision [4]. The CRLB is based on the Fisher information matrix [12], which in turn makes use of the second derivative of the location posterior distribution with respect to the location parameters. However, Wi-Fi fingerprinting gives rise to a discrete classification problem, i.e., find the discrete location from a finite set of reference locations that fits the measurements best. Hence, as illustrated in Figure 1, these posterior distributions cannot be differentiated with respect to the location parameters, as they are discrete variables. The dilution of precision is calculated based on the geometry of transmitters and receivers [13]. In our data, we have no information of the location of the transmitters, making this approach unfeasible. The conditional entropy, on the other hand, can be calculated for any posterior distribution over the reference locations based on a single Wi-Fi measurement. Note that while our focus is on Wi-Fi fingerprinting, our results apply to any probabilistic localization system that calculates a posterior probability distribution of the form P(Pos∣m) , where Pos is a finite set of locations in the environment and *m* is a measurement that serves as input for the localization system. This type of localization occurs in applications where topological maps are more appropriate than metric ones, such as localization of tags fixed to medical equipment in a hospital setting. Learning that a particular piece of equipment is localized in a particular room without knowing its exact position is sufficient for retrieval and arguably more useful than learning that it is at a precisely-defined position that may turn out to be physically unreachable.

This paper continues as follows. We first discuss our probabilistic sensor model and its three different training options: an incorporation of the uncertainty resulting from unknown antenna gain, using a trained or fixed standard deviation and using a single location for training or a small region of locations. We also discuss the calculation of the conditional entropy, the setup of the datasets and how the experiments are conducted. We then present the three sets of location error and conditional entropy results. We discuss the results in the following section. Finally, we provide our conclusion and future work.

## 2. Methods

We propose that there is a relation between the error on the location estimate and the conditional entropy in the posterior probability distribution over the locations. Both metrics quantify the uncertainty on the location estimate: the location error as a region of possible locations and the conditional entropy as the average Shannon information content [14].

To be able to calculate the conditional entropy, we created a localization algorithm using a probabilistic Wi-Fi fingerprinting sensor model. The sensor model must be probabilistic because the conditional entropy can only be calculated from a posterior probability distribution. The Wi-Fi fingerprinting sensor model allows us to use large open access datasets for our experiments. The experiments consist of calculating the location error of the algorithm given a single measurement, calculating the conditional entropy given the same measurement and studying the relation between the two.

First, we describe our probabilistic sensor model. Second, we detail how the sensor model can be trained. Third, we introduce the formula for conditional entropy. Fourth, we discuss the open access Wi-Fi fingerprinting datasets. Finally, we describe the experiments.

### 2.1. Sensor Model

Wi-Fi fingerprinting is a frequently-used approach for indoor localization [15,16]. Generally, Wi-Fi fingerprinting consists of two phases: an offline training phase and an online localization phase. During the training phase, Wi-Fi fingerprints are measured at specific reference locations and stored in a database. During the localization phase, a new Wi-Fi fingerprint is compared to the database to indicate the most likely location at which the new fingerprint is measured [17].

In any environment, there is a list of *n* access points that are received somewhere in the environment: $A=(a_{1},a_{2},\ldots ,a_{n})$ . This list can include each access point, even if some are received only once at a single reference location. The list can also be more restrictive, for example by selecting only access points that are installed by the researchers or only those access points that are received with a minimum received signal strength [18]. We use the first option, a list of all of the access points that have been received.

A fingerprint is the received signal strength, often indicated by its received signal strength indicator (RSSI) value, at a location for these access points *A*: $\vec{w}=\left(w_{1},w_{2},\ldots ,w_{a},\ldots ,w_{n}\right)$ . The value of $w_{a}$ can be any value or can be empty, if the access point is not received at that location in the environment. Not receiving an access point can be caused by interference, obstruction of the Wi-Fi signal or because the distance between the measurement location and the access point is too large; this occurs more often if the environment is complex or large.

During the training phase, a set of Wi-Fi fingerprints $W_{t}$ is measured at reference locations and stored in a database, which we call the training database. This set of fingerprints can be used directly as the representation of the reference location, with each individual measurement as a representation of the same location. Such an approach is often used in signal space *k*-NN localization algorithms [19,20,21]. Alternatively, the set of fingerprints can be used to create a template of the Wi-Fi signal at the reference location, using for example the average and variance of the RSSI values or selected percentiles of the RSSI values [22]. Finally, the set of access points in a fingerprint can also be extended by the pairwise difference of the RSSI values [23], after which the above approaches can be applied to the extended fingerprint.

During the localization phase, a new Wi-Fi fingerprint is compared to the training database. This comparison is a distance metric, such as the Euclidean distance, in the case of signal space *k*-NN. The comparison can also be a likelihood function, if a probabilistic model is created from the training data $W_{t}$ at each reference location. After the comparison, the *k*-NN algorithm will select the *k* reference locations with the smallest distance metric and select the average location as the location estimate; some variants calculate a weighted average. The probabilistic approach will calculate the posterior probability distribution of the locations based on the calculated likelihoods and a given prior distribution. Minimizing a loss function on the posterior distribution results in a location estimate. We choose to use the error probability loss function, which translates into selecting the reference location with the maximum value in the posterior distribution. We use a probabilistic sensor model, so that we can calculate the uncertainty in the posterior distribution.

Depending on whether an access point is received in the fingerprints of the template and the new fingerprint, there are four possible situations: a hit, when the access point is visible in both the template and the new fingerprint, a miss, when the access point is visible in the template, but not in the new fingerprint, an extra, when the access point is visible in the new fingerprint, but not in the template, and a none, when the access point is not visible in either the template or the new fingerprint. Table 1 shows an example of these situations.

The relevant literature usually explains how to deal with the hit case, but is seldom clear how to deal with the miss, extra, and none cases. Sometimes, the paper only shows results of experiments where each access point is received everywhere in the environment [23,24,25]. In other cases, the RSSI of access points that are not received are set to the minimal possible RSSI value [18] or cause a penalty in the comparison process [8,17]. We design our sensor model so that a likelihood can be calculated at each reference location for all access points.

For convenience, we assume that the received signal strengths of the different access points are independent, which is a form of the naive Bayesian, resulting in the formula for the likelihood at a specific location pos being:

(1) $$P\left(\vec{w}|pos\right)=\prod_{a=1}^{n}P(w_{a}|pos).$$

**Figure 2:**
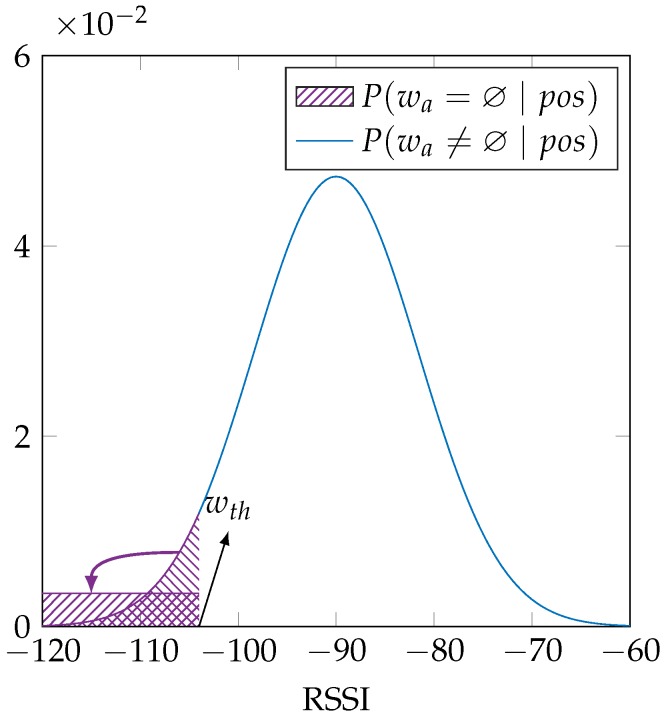
Our sensor model explicitly allows an access point to be not received, indicating it as having an RSSI value lower than the threshold RSSI value $w_{th}$ .

For each access point *a* in the fingerprint, any of the four situations of Table 1 is possible. In these situations, we observe two possibilities for any RSSI measurement of $w_{a}$ at location pos : either it has a value or the access point is not received at that location. In a training dataset of Wi-Fi fingerprints, there is a minimal RSSI value, which we call the threshold value $w_{th}$ : we can say that when an access point is not received at location pos , its RSSI value is any value lower than this threshold $w_{th}$ . Therefore, we model the RSSI values as a uniform distribution for values lower than the threshold and model them as a normal distribution for values higher than the threshold. The density in the tail of the normal distributions of values below $w_{th}$ is redistributed as a uniform distribution for these values below $w_{th}$ . Figure 2 shows this dual distribution, which is mathematically equal to:

P(wa∣pos)={(2a)1wth−wmin∫wminwthN(w∣μa,σa2)dw,for wa=∅,(2b)∫wa−0.5 dBwa+0.5 dBN(w∣μa,σa2)dw,for wa≠∅,

where $N(w|\mu ,\sigma^{2})$ is the normal distribution with mean *μ* and variance $\sigma^{2}$ ; $\mu_{a}$ and $\sigma_{a}^{2}$ are functions of pos and are calculated for each reference location in each dataset separately; $w_{th}$ is our threshold RSSI value, which is also specific to each dataset; $w_{min}$ is our lower bound RSSI value, so that we can numerically calculate the likelihood; and 1 dB is the typical measurement precision of Wi-Fi RSSI measurements.

**Figure 3:**
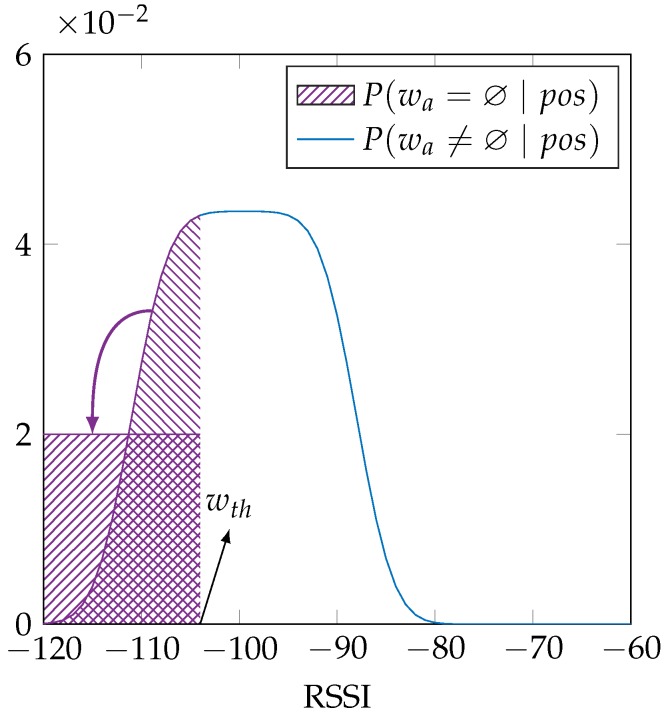
The extension of our sensor model incorporates uncertainty on the antenna gain, by a convolution with a uniform distribution.

The RSSI value also depends on the antenna gain, which can be different in different antenna poses at the same location. These differences, when included in the training data, will cause an increased variance of the sensor model. When the differences are not included in the training data, the sensor model may result in a low likelihood for a measurement from a location near or at the reference location, but at a different antenna pose. This uncertainty about the antenna pose can be modeled as a uniform distribution between the minimum and maximum antenna gain, $g_{min}$ and $g_{max}$ . We propose a second probabilistic sensor model by introducing this uniform distribution into our first sensor model. We argue that the use of the uniform distribution is justified because it incorporates the effect of the antenna gain, in the absence of any information on the antenna gain, such as the antenna properties, pose variation and multipath conditions. Alternatively, one could argue that a normal distribution is a better choice when there are several independent factors contributing. As tests showed that the actual entropy values resulting from using a normal distribution depend on its chosen width and as we could not find any relevant data to choose this width for our experiments, we have chosen to use the uniform distribution, as this introduces less assumptions into the modeling, i.e., a maximum entropy argument regarding this aspect of the model. This is achieved by a convolution of the uniform distribution with the sensor model [26], which results in the distribution in Figure 3 and is mathematically:

(3) $$P\left(w_{a}|pos\right)=\frac{1}{g_{max}-g_{min}}\int_{g_{min}}^{g_{max}}P\left(w_{a}-g|pos\right)dg,$$

where $P(w_{a}-g|pos)$ is given by Equation (2). We select the constant values of $g_{max}$ and $g_{min}$ from antenna specifications.

These Wi-Fi sensor models do not fully incorporate the situation where an access point is not received due to interference. With destructive interference, caused by multipath reception of the access point’s signal, as described by Phillips et al. [27], the RSSI value will decrease, potentially prohibiting the reception of the signal. This kind of interference is captured by the sensor model, since in that situation, the RSSI value will be lower than the threshold $w_{th}$ . However, the channel may become too busy, such as described by Liang et al. [28]. While Wi-Fi will try to avoid transmission when the channel is busy by using channel sense multiple access with collision avoidance [29], heavy traffic may prohibit that the Wi-Fi access point is received at a location where it is normally received. This is currently not incorporated in the sensor model. However, this is not the default condition in Wi-Fi networks, and we assume that the datasets that we use are not collected in such conditions.

### 2.2. Training the Sensor Model

Both implementations of our sensor model depend only on the parameters *μ* and *σ*. These must be trained for each access point at each reference location, but we cannot use the familiar approaches for calculating the mean and variance, since those formulas are not designed to include the part with the uniform distribution. In other words, they cannot deal with empty values, such as those from access points that are not received in the training data.

However, we can calculate the likelihood of the parameters for a given RSSI value $w_{a}$ using a constraint optimization. We create a range of feasible *μ* and a range of feasible *σ* values. These constraints are between −130dBm and −30dBm for *μ* and between 1dBm and 30dBm for *σ*. We then calculate the likelihood of those parameters using Equation (2a) if $w_{a}$ has no value (the access point is not received) and Equation (2b) if $w_{a}$ has a value. We calculate these likelihoods of the parameters for all *w* values of access point *a* at reference location pos , so that we can multiply the likelihoods over the *w* values:

(4) $$\mu_{a},\sigma_{a}=\underset{\mu ,\sigma }{\arg\max}\prod_{w\in W_{t,a,pos}}P\left(w|\mu ,\sigma \right),$$

where $W_{t,a,pos}$ is the set of all *w* values in the training dataset *t* for access point *a* at reference location pos and P(w∣μ,σ) can be calculated as in Equation (2). The pair of *μ* and *σ* parameters that maximizes this likelihood is then selected as the parameters for access point *a* at location pos . The training is analogous for the sensor model that incorporates the antenna gain in Equation (3).

While the value of *μ* is usually satisfactory after training, the value of *σ* can be rather small. This is due to the training procedure, which must balance between received and not received access points, given a limited amount of data. When but a small fraction of the samples have received the access point, the procedure tends to increase *μ* before increasing *σ*. Therefore, we will not only include results using the trained *σ* as described above, but also using a fixed *σ* for all access points at all reference locations. The fixed *σ* is the median value of *σ* of all reference locations and access points, when the access point is received at least once at the reference location.

**Figure 4:**
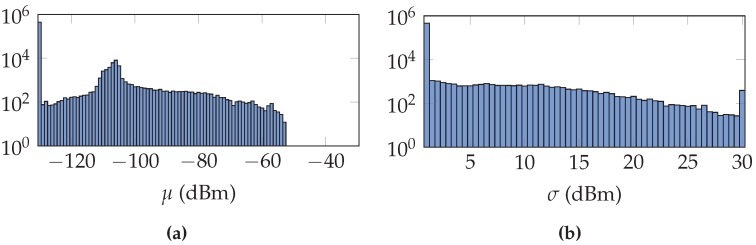
The histograms of parameters *μ*, (**a**) and *σ*, (**b**) after the training procedure in the UJIIndoorLoc dataset, with the uncertainty of the antenna gain incorporated and using a region of reference locations.

Finally, the number of reference locations in an environment is always limited. Two reference locations that are nearby can thus have very distinct values for $\mu_{a}$ and $\sigma_{a}$ , while a reference location much further away can by chance match one of the two locations better. To overcome this issue, we propose to use as training data not just the data from a single reference location, but also to include the data from the four nearest reference locations. Given a Wi-Fi fingerprint to localize, we want to increase the likelihood in a region near the correct reference location and decrease the influence of a further away reference location that could match the fingerprint by chance. Figure 4 is an example of the distribution of the parameters $\mu_{a}$ and $\sigma_{a}$ after training in the UJIIndoorLoc dataset with the uncertainty of the gain incorporated and using a region of reference locations.

### 2.3. Conditional Entropy

As a dynamic metric for the uncertainty in the localization system’s sensor model, we propose the conditional entropy, a quantity from information theory. The entropy of a probability distribution is defined as the average Shannon information content, where the Shannon information content expresses how much is learned by observing a specific outcome [14,30]:

(5) $$H\left(X\right)\equiv \sum_{x\in X}P\left(x\right)\log\frac{1}{P(x)},$$

where *X* is a set of possible outcomes *x* and P(x) is the probability assigned to outcome *x*; from which follows the conditional entropy; see Cover [30]:

(6) $$H\left(X|y\right)\equiv \sum_{x\in X}P\left(x|y\right)\log\frac{1}{P(x|y)}.$$

In our setup, *X* is the set of possible locations, i.e., the reference locations in the training dataset, Pos . The posterior distribution of the locations is conditional on the measurement, $y\mapsto \vec{w}$ , in our case a Wi-Fi fingerprint. Substituting, the equation becomes:

(7) $$H\left(Pos|\vec{w}\right)=\sum_{pos\in Pos}P\left(pos|\vec{w}\right)\log\frac{1}{P(pos|\vec{w})},$$

where we can calculate the posterior distribution of the location pos given the measurement *w* using the likelihood calculated in Equation (1) and applying Bayes’ rule:

(8) $$P\left(pos|\vec{w}\right)=\alpha P\left(\vec{w}|pos\right)P\left(pos\right),$$

where *α* is a normalization constant and P(pos) is assumed to be a uniform distribution, since we are studying the localization of a single measurement.

**Figure 5:**
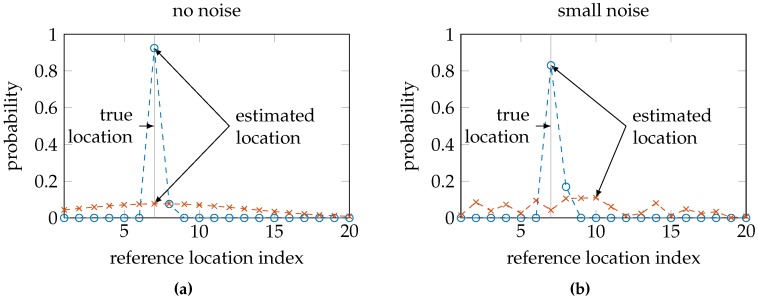
A small amount of white noise has little influence on the probability mass function with little uncertainty (the blue circles); the noise has much more influence on the function with high uncertainty (the red crosses). The dashed connecting lines have been added as visual aids. We want to know if low uncertainty also corresponds with low location error.

The entropy expresses the uncertainty in a probability distribution. Distributions indicating high uncertainty will have a high entropy, such as a uniform distribution or a normal distribution with high variance. On the other hand, distributions indicating high certainty will have low entropy, such as a normal distribution with low variance or a distribution that resembles a Dirac’s delta function. Consequently, a sensor model that sufficiently captures the uncertainties in the environment should result in a posterior probability distribution with a small location error when the conditional entropy is low and a high conditional entropy when the location error is large; see also Figure 5.

The conditional entropy can be calculated for any posterior distribution, so this approach is not restricted to Wi-Fi fingerprinting alone. Any localization system that has a probabilistic sensor model can calculate the uncertainty in the posterior distribution as the conditional entropy.

### 2.4. Data

We use three very different, publicly-available datasets. These datasets all have a comparable setup: all provide indoor Wi-Fi fingerprints at a number of reference locations. They are created in very different environments. One dataset is created in the w-iLab.t II testing environment, which is a wireless communication testing facility located above a clean room [9]. It is part of the EVARILOS localization benchmarking project, hence the label we use for the dataset. It contains many metal features, making it a worst case scenario for Wi-Fi fingerprinting localization. However, there are more Wi-Fi access points than in a typical environment. A second dataset is created at the GEOTEC Lab [10]. This environment is a laboratory and office floor. A third dataset is created in the university buildings of the Universitat Jaume I (UJI) [11]. It is a very large dataset, covering three different buildings with up to five floors.

The data are used in separate training and validation sets. The GEOTEC and UJIIndoorLoc datasets provide this separation themselves. For the EVARILOS dataset, we selected 10% of the reference locations and used all fingerprints at these locations as validation data, of course excluding them from the training data. By using all fingerprints at these locations, we made sure that the performance of the system is not falsely improved by having trained at the exactly correct location. The number of fingerprints used for both training and validation are shown in Table 2. This table also includes the total number of AP in the dataset, the number of reference locations, the total area covered by the dataset and the maximum conditional entropy corresponding with the number of reference locations in these environments.

### 2.5. Experiments

Our experiments consist of applying our sensor model to the data from a validation dataset, after training its parameters using a training dataset. After applying our sensor model to a measurement from the validation data, we obtain the posterior probability distribution over the reference locations. From this distribution, we derive two results: the location error and the conditional entropy. Subsequently, we can study the relation between these metrics.

The location error is calculated as the Euclidean distance between the true location at which the validation measurement was performed and the location estimated based on the posterior distribution. In the UJIIndoorLoc dataset, which features buildings with up to five floors, we used a constant floor height of four meters. To derive this location estimate, we select the reference location with the highest posterior probability. This approach is called the maximum a posteriori location estimate. As discussed before, Wi-Fi fingerprinting is a form of classification, selecting this location estimate from the set of reference locations learned during training. Other estimators may result in location estimates that are not valid or reachable in the environment. While such anomalies can be corrected by additional post-processing of the location estimate, we specifically want to look into the relation between the conditional entropy and the location error as derived from the sensor model without further information.

The conditional entropy is calculated as discussed earlier.

We propose that a high quality sensor model is one for which validation samples that produce a posterior probability distribution with low conditional entropy also have a low location error. Analogously, validation samples that receive a location estimate with a large location error should also have produced a posterior distribution with high conditional entropy. It is possible that a validation sample has a small location error and a high conditional entropy, since the reference location with the highest posterior probability can still be the correct or nearly the correct location. It should not be possible that a validation sample has a large location error with a low conditional entropy: this would indicate a high certainty in the posterior distribution, while the estimate is still at a large distant from the correct location.

**Figure 6:**
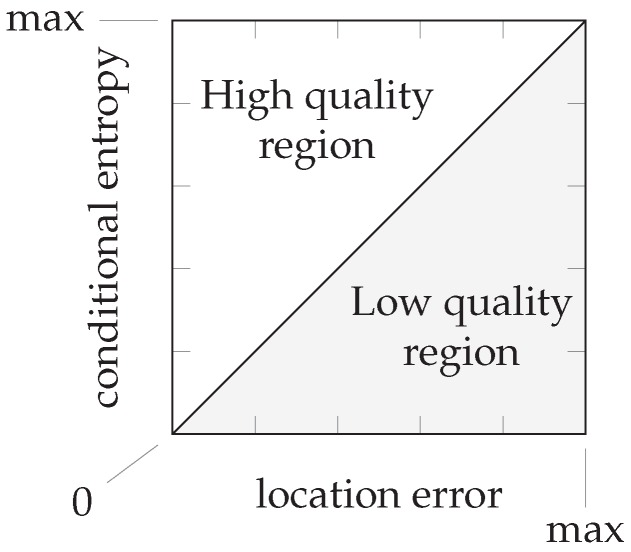
An estimate of a perfect linear relationship between the location error and conditional entropy divides the space of the two metrics into halves: we say that the upper triangle is the region where samples of high quality sensor models can be found and the lower triangle is the region where samples of low quality sensor models can be found. This allows us to calculate a quality measure, although it is only indicative. We are especially interested in the border cases, with very small conditional entropy and very large location error.

If we plot the validation samples’ conditional entropy versus their location error, we obtain a scatter plot in a diagram such as the one in Figure 6. This entropy should relate to the error in the location estimate. In particular, we expect that a very uncertain distribution is more likely to have an inaccurate location estimate, and a very certain distribution is more likely to have an accurate location estimate (in accordance with Figure 5). As an estimate of a perfect linear relation between the location error and conditional entropy, we draw a line through the origin and the point with maximum conditional entropy and maximum location error. The maximum conditional entropy depends solely on the number of reference locations because it is achieved when the probability over all reference locations is equal. The maximum location error is chosen as the maximum distance between any two reference locations in the training database. When a validation sample has a location error and conditional entropy that lies above this line, we say that the sensor model produced a sufficiently uncertain distribution given the measurement; when it is below the line, we say that the sensor model produced a distribution with false certainty given the measurement. Since small location errors can result from both uncertain and certain distributions centered on the correct location and large errors should only result from uncertain distributions, samples produced by a high quality sensor model are expected to fall within the upper triangular region. We determine a quality measure as the ratio of samples within the upper triangular region on the total number of validation samples. This quality measure is but an approximation to aid the discussion, since we do not know the true relationship. We are especially interested in the validation samples with very low conditional entropy, but a significant location error.

## 3. Results

The location error and conditional entropy results are presented separately for each dataset: first, the EVARILOS dataset, then the GEOTEC dataset and, finally, the UJIIndoorLoc dataset. Both metrics are displayed as a cumulative distribution and as a summarizing table. The summarizing table lists the mean, standard deviation, median and 95th percentile. The 95th percentile is the location error so that 95% of the validation samples have an equal or lower location error. Then, we present a table of the quality measure. Finally, we illustrate the correlation by scatter plots of selected datasets and sensor model setups.

Eight different sensor model setups are discussed, so that we have a combination of each of the three different options in the sensor model. The selected option is indicated by a single letter, so that the combination can be indicated with a label of three letters: either with (g) or without (n) incorporating the uncertainty resulting from the unknown antenna gain, using a trained *σ* (t) or a fixed *σ* (f) and using a single reference location’s set of data (s) or a region of five reference locations (r).

**Figure 7:**
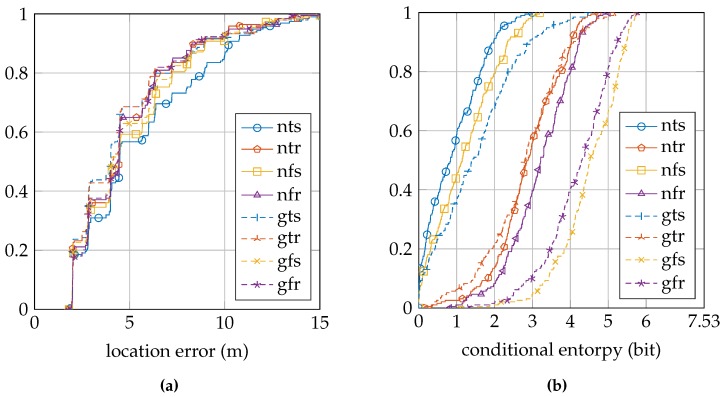
EVARILOS cumulative distributions: (**a**) location error; (**b**) conditional entropy. The legend labels refer to using the normal sensor model (n) or using the convolution sensor model (g), using the trained *σ* (t) or using the fixed *σ* (f) and using a single reference location for training (s) or using a region of reference locations for training (r).

Table 3 summarizes the location error and conditional entropy results for the EVARILOS dataset. Figure 7a shows the cumulative distribution of the location error, and Figure 7b shows the cumulative distribution of the conditional entropy.

**Figure 8:**
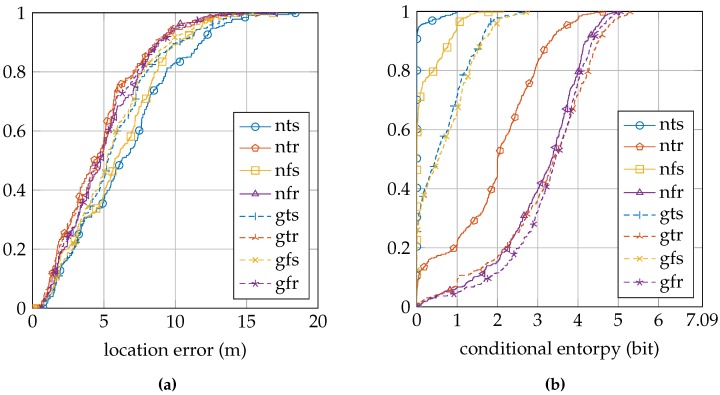
GEOTEC cumulative distributions: (**a**) location error; (**b**) conditional entropy. The legend labels refer to using the normal sensor model (n) or using the convolution sensor model (g), using the trained *σ* (t) or using the fixed *σ* (f) and using a single reference location for training (s) or using a region of reference locations for training (r).

Table 4 summarizes the location error and conditional entropy results for the GEOTEC dataset. Figure 8a shows the cumulative distribution of the location error, and Figure 8b shows the cumulative distribution of the conditional entropy.

**Figure 9:**
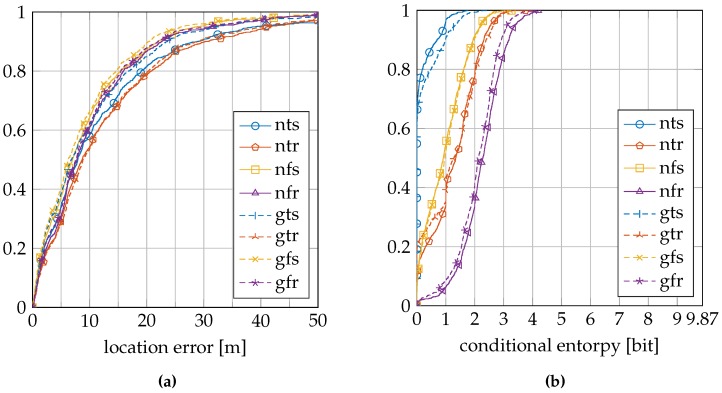
UJIIndoorLoc cumulative distributions: (**a**) location error; (**b**) conditional entropy. The legend labels refer to using the normal sensor model (n) or using the convolution sensor model (g), using the trained *σ* (t) or using the fixed *σ* (f) and using a single reference location for training (s) or using a region of reference locations for training (r).

Table 5 summarizes the location error and conditional entropy results for the UJIIndoorLoc dataset. Figure 9a shows the cumulative distribution of the location error, and Figure 9b shows the cumulative distribution of the conditional entropy.

Table 6 shows the percentage of validation samples in the high quality region. This fraction of the samples has a low conditional entropy only when the location error is low and have a higher conditional entropy as the location error increases; see also Figure 6.

**Figure 10:**
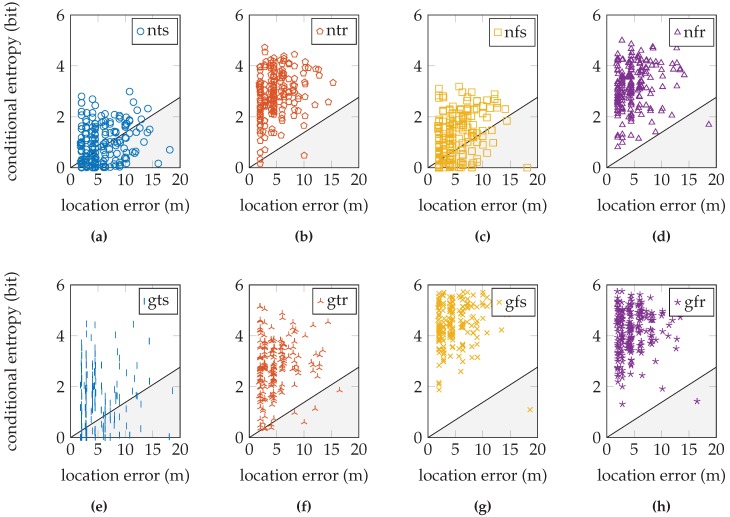
The relation tested on the EVARILOS dataset. The legend labels refer to using the normal sensor model (n) or using the convolution sensor model (g), using the trained *σ* (t) or using the fixed *σ* (f) and using a single reference location for training (s) or using a region of reference locations for training (r).

**Figure 11:**
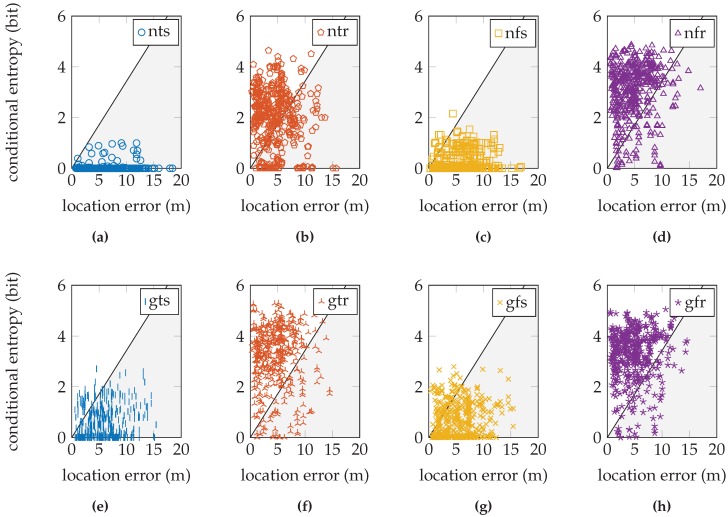
The relation tested on the GEOTEC dataset. The legend labels refer to using the normal sensor model (n) or using the convolution sensor model (g), using the trained *σ* (t) or using the fixed *σ* (f) and using a single reference location for training (s) or using a region of reference locations for training (r).

**Figure 12:**
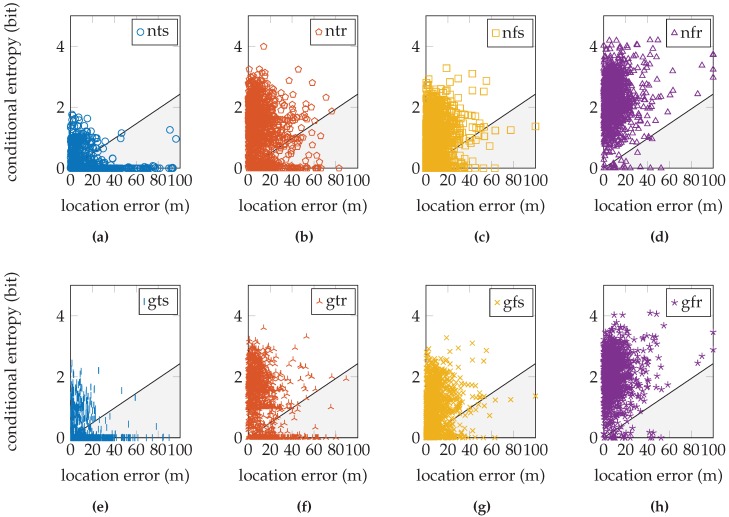
The relation tested on the UJIIndoorLoc dataset. The legend labels refer to using the normal sensor model (n) or using the convolution sensor model (g), using the trained *σ* (t) or using the fixed *σ* (f) and using a single reference location for training (s) or using a region of reference locations for training (r). Some samples have a location error greater than 100 m. These have been drawn on the right-hand vertical axis; all of these samples are actually below the diagonal.

These fractions are shown graphically in Figure 10 for the EVARILOS dataset, Figure 11 for the GEOTEC dataset and Figure 12 for the UJIIndoorLoc dataset.

## 4. Discussion

The relation between the conditional entropy and the location error, as seen in Figure 10, Figure 11 and Figure 12, is how we propose to study the quality of the sensor models. The estimate of the quality measure guides in finding sensor model implementations of higher quality; see Table 6 for these measures. We observe that within a dataset, the lowest measure is obtained with the most basic setup of the sensor model, the model that does not incorporate the antenna gain, that uses a trained *σ* and that is trained at a single reference location (nts). The conditional entropy is usually low or even zero when using this approach; see Figure 7b, Figure 8b and Figure 9b; while the location error can be among the largest of any sensor model approach within the dataset; see Figure 7a, Figure 8a and Figure 9a. We would expect the validation samples with low conditional entropy to have a small location error. However, this is contradicted when studying the relation between the two metrics in Figure 10a, Figure 11a and Figure 12a. Validation samples with very low conditional entropy can still have a relatively large location error using this basic sensor model approach.

Changing the sensor model often leads to an increased conditional entropy and not necessarily to an increased location error. We will discuss the three different choices made when building the sensor model one by one.

Firstly, we can change the sensor model approach by using a fixed value for the parameter *σ* instead of training it individually for each access point at each reference location. The results of this approach change from a label with the letter ”t”to the letter ”f”. In the EVARILOS dataset, using such a fixed *σ* instead of a trained *σ* has little influence on the location error, but increases the conditional entropy; see Table 3. In the GEOTEC dataset, it almost always decreases the location error and always increases the conditional entropy; see Table 4. In the UJIIndoorLoc dataset, it always decreases the location error and increases the conditional entropy; see Table 5.

Using this fixed *σ* instead of a trained *σ* has the most effect when the environment contains extra access points, which are access points that are not or only rarely received during training, but can be received at the reference location during the localization phase, as explained in Table 1. Our training procedure will select the lowest possible parameters for both *μ* and *σ* for an access point that is not received during training, effectively placing all of the probability density of the sensor model under the threshold value $w_{th}$ as indicated in Figure 2 and Figure 3. The probability of a reference location for which a validation sample has an extra access point will become very low, even though it may be the correct reference location. Such a very low probability is avoided when using a fixed value for parameter *σ*, a value that is larger than the minimal feasible value, since this will push some of the probability density above the threshold $w_{th}$ . This is what happens in the GEOTEC and UJIIndoorLoc datasets, which contain many access points, of which only a few are received often in the training samples at a single reference location.

Using a fixed parameter *σ* has little effect on the location error in the EVARILOS dataset, because in this dataset, all access points are received at all reference locations, for almost all training samples. The increase in conditional entropy is most visible when incorporating the uncertainty resulting from the unknown antenna gain. This convolution with a uniform distribution already introduces a large variance in the sensor model, so the trained parameter *σ* will typically be lower, creating very steep slopes in the sensor model, which causes a quickly decreasing likelihood to be assigned to access points that are received at a RSSI value just outside the antenna gain range. Selecting a fixed, larger parameter *σ* prevents the sensor model from quickly assigning a very low likelihood.

Secondly, we can change the sensor model approach by using a region of reference locations for training rather than a single reference location. The results of this approach change from a label with the letter ”s” to the letter ”r”. Applying this approach increases the conditional entropy in all datasets. The increase in conditional entropy is expected, since a small region of locations will have a comparable model of the Wi-Fi signal. The exception is the EVARILOS dataset when using a fixed *σ* and the antenna gain is incorporated; see Table 3. The conditional entropy in this dataset is already relatively high when using a single reference location, fixed *σ* and the antenna gain. The randomness of the Wi-Fi signal that is modeled in that approach is not increased by training at a region of reference locations.

Using a region of reference location to train the sensor model increases the location error in the UJIIndoorLoc dataset; see Table 5. The dataset has a sparsely distributed set of reference locations, which causes the regions of reference locations to be contained in small sets, like little islands of reference locations. Especially the validation samples that are collected outside these sets of reference locations have an increased location error, because the model of the Wi-Fi signal within the set is quite different from the Wi-Fi signal outside the set. The reference locations closest to the locations of the validation samples outside the sets are those reference locations at the borders of these sets. Their sensor model parameters will be influenced by the training data of the reference locations within their region, inside the set, which is further away from the validation samples; thus, the sensor model is likely to be a worse model for the validation samples outside the set, which is not the case when we train the sensor model at only a single reference location.

Using this region of reference locations for training decreases the location error in the GEOTEC dataset, which has a more densely distributed set of reference locations. The same formation of sets of these regions as in the UJIIndoorLoc exists, with validation samples outside of these sets having a larger location error. However, this is compensated by the effect that the Wi-Fi signal is better modeled within the regions of reference locations, especially a reduction in extra access points.

Thirdly and lastly, we can incorporate the antenna gain by performing a convolution with a uniform distribution on the range of the antenna gain, as illustrated in Figure 3. The results of this approach change from a label with the letter ”n” to the letter ”g”. The conditional entropy typically increases when applying this change in the EVARILOS and GEOTEC datasets, as expected. The conditional entropy changes only very slightly in the UJIIndoorLoc dataset. This dataset has the largest signal variance due to hardware heterogeneity during the training phase, increasing the parameter *σ* and decreasing the impact of the convolution with the uniform distribution.

Note that the location error in Figure 7a never reaches zero. This is caused by our selection of 10% of the training data as validation data and because we use the maximum a posteriori location estimation. The former reason ensures that the true validation sample’s location is never exactly at a reference location. The latter reason allows only reference locations to be location estimates, so estimating the exactly correct location is impossible in this usage of the dataset.

Summarizing, if the environment has a dense distribution of reference locations and the visible access points at a reference location are received often in the training data, as in the EVARILOS dataset, using a trained value for parameter *σ* is the best approach. Additionally, incorporating the antenna gain and training at a region of reference locations enable a better model of the Wi-Fi signal’s randomness, which decreases the location error. Figure 10f shows the relation between the conditional entropy and location error for the EVARILOS dataset that uses this sensor model approach. The sample cloud forms a tail towards the lower left-hand corner; low conditional entropy corresponds with small location error. This is one of the higher quality sensor model implementations in the EVARILOS dataset and achieves a measure of 95.9 %. A high measure is also achieved by the same sensor model without the antenna gain; see Figure 10b. This is also a high quality sensor model, but has a slightly larger median location error: 4.44 m from 4.06 m . The sensor model approach with antenna gain, a fixed *σ* and trained at a single reference location in Figure 10g also has a small location error and high conditional entropy and also shows the tail towards the lower left-hand corner even with a quality measure of 99.5 %. However, the relation diagram shows that the conditional entropy has increased too much. This should be avoided, as it indicates that too much uncertainty has been introduced in the sensor model, i.e., the sensor readings are assumed less reliable than they really are. In this case, the same hardware is used throughout both the training and the validation data collection, both performed with high repeatability using a robot. As these factors limit antenna gain uncertainty, this explains why the models without antenna gain compensation already perform quite well. Figure 10e shows that not enough uncertainty is included in the sensor model that incorporates the antenna gain, has a trained *σ* and is trained at a single reference location. Many validation samples have little or zero conditional entropy, while some of them have a relatively large location error. Even though this sensor model approach achieves the smallest location error in the EVARILOS dataset, we do not think it is the best approach for the environment, since the posterior probability distribution may display an incorrect certainty.

When the environment has a dense distribution of reference locations, but there are access points that are received only seldom at a reference location, as in the GEOTEC dataset, it is better to use a fixed value for parameter *σ* in our sensor model training approach. Again, incorporating the antenna gain and training at a region of reference locations allows a smaller location error. Figure 11h shows the relation between the conditional entropy and the location error for the GEOTEC dataset that uses this sensor model approach. We expect the validation sample cloud to be similar to the one from the EVARILOS dataset, especially the tail towards the lower left corner, which would indicate that lower conditional entropy corresponds with lower location error. The sample cloud’s shape does show more similarity with the expected tail than the shapes of the sample clouds with lower conditional entropy, such as in Figure 10a,c,e,g; thus, it seems that we do not model enough uncertainty in the sensor model approach. When we include more uncertainty in the sensor model, the sample cloud seems to rise, revealing more of the expected tail, as happens in Figure 10b,d,f,h.

The same is true for the UJIIndoorLoc dataset, where in any approach, there remain validation samples with very little or zero conditional entropy; see Figure 12. This means that the posterior probability distribution indicates a single reference location as the location where the validation sample was created and applies no probability whatsoever to any other reference location. This is unlikely in Wi-Fi fingerprinting, where similar fingerprints can be found at different locations. This certainty in the posterior distribution is caused by the sensor model. Consequently, we know that we do not yet model enough uncertainty in the sensor model. The GEOTEC and UJIIndoorLoc datasets are both created with heterogeneous hardware, which may be the cause of the extra randomness that is not included in the sensor model.

## 5. Conclusions

We have studied the uncertainty of a Wi-Fi fingerprinting localization system by relating the location error with the conditional entropy in the location posterior probability distribution. We calculated the location error and conditional entropy in three different Wi-Fi fingerprinting datasets, using eight variations of a probabilistic sensor model. We proposed that a high quality sensor model has a small location error when the conditional entropy is low and a high conditional entropy when the location error is large. A low quality sensor model is one that has a large location error, but low conditional entropy, so that the posterior probability distribution has a high certainty, but actually leads to a wrong location estimate. We also calculated the fraction of validation samples processed by the sensor model that complied with a rough estimate of this relationship as an estimate of the sensor model’s quality.

We find that the median conditional entropy increases when we model more of the environment’s uncertainty in the sensor model, without necessarily increasing the median location error, indeed sometimes decreasing the median location error. The additional sources of uncertainty in the sensor model that we study are the antenna gain, a fixed, generally larger parameter *σ* and increasing the region of reference locations used for training. As the median conditional entropy increases for different implementations of the sensor model, we see that samples that generate a low conditional entropy tend to have a low location error. Additionally, samples that generate a high conditional entropy can have either a low or high location error. Thus, these implementations are increasingly higher quality sensor models. Of course, even better Wi-Fi fingerprinting sensor models may exist. One source of uncertainty that is not yet included in the model is the absence of an RSSI value of an access point due to interference in the signal.

With this information, we can start to create probabilistic sensor models that not only estimate the location, but can also correctly indicate the uncertainty of the location estimate. This is especially useful in applications where the reliability of the location estimate is of equal importance as the location error. Such applications include reducing the resources required by the localization system, such as energy and processing time, when a sufficiently reliable location estimate is achieved. More broadly, creating a high quality sensor model in the sense that we defined in this paper allows for the correct assessment of the uncertainty over the location estimate without requiring a ground truth.

## Figures and Tables

**Table 1:** The relevant literature is not always clear how to apply the sensor model on training data ${\vec{w}}_{t}$ and measurement ${\vec{w}}_{f}$ when some access points are not received (∅) in either of the two fingerprints.

*A*	${\vec{w}}_{t}$ (dBm)	${\vec{w}}_{f}$ (dBm)	Situation
$a_{1}$	−60.0	−59.0	*hit*
$a_{2}$	−89.0	∅	*miss*
$a_{3}$	∅	−74.0	*extra*
$a_{4}$	∅	∅	*none*

**Table 2:** Overview of the datasets with $N_{t}$ fingerprints used for training, $N_{v}$ fingerprints used for calculating the results, AP access points, Pos reference locations, *A* the total floor area and $H_{max}$ the maximum conditional entropy. UJI, Universitat Jaume I.

Name	Location	References	$N_{t}$	$N_{v}$	AP	Pos	$A\;(m^{2})$	$H_{max}\;\left(\mathrm{bit}\right)$
EVARILOS	w-iLab.t II	[9]	1800	194	44	185	1302	7.53
GEOTEC	GEOTEC Lab	[10]	860	460	97	136	207.48	7.09
UJIIndoorLoc	UJI	[11]	20	1111	520	933	108.70	9.87

**Table 3:** EVARILOS summarizing table. The column labels refer to using the normal sensor model (n) or using the convolution sensor model (g), using the trained *σ* (t) or using the fixed *σ* (f) and using a single reference location for training (s) or using a region of reference locations for training (r).

Error (m)	nts	ntr	nfs	nfr
mean	5.65	4.91	5.15	4.90
standard deviation	3.39	2.97	3.03	2.93
median	4.47	4.44	4.45	4.44
95th percentile	11.99	10.20	11.22	11.56
	**gts**	**gtr**	**gfs**	**gfr**
mean	4.78	4.71	4.98	4.86
standard deviation	3.16	2.96	3.02	2.82
median	4.00	4.06	4.06	4.36
95th percentile	11.65	11.65	10.77	11.02
**Entropy (bit)**	**nts**	**ntr**	**nfs**	**nfr**
mean	0.92	2.88	1.20	3.19
standard deviation	0.75	0.88	0.85	0.86
median	0.84	2.87	1.15	3.22
95th percentile	2.19	4.18	2.73	4.40
	**gts**	**gtr**	**gfs**	**gfr**
mean	1.50	2.78	4.50	4.19
standard deviation	1.08	1.04	0.84	0.89
median	1.43	2.80	4.52	4.30
95th percentile	3.49	4.50	5.58	5.44

**Table 4:** GEOTEC summarizing table. The column labels refer to using the normal sensor model (n) or using the convolution sensor model (g), using the trained *σ* (t) or using the fixed *σ* (f) and using a single reference location for training (s) or using a region of reference locations for training (r).

Error (m)	nts	ntr	nfs	nfr
mean	6.50	4.69	5.93	5.02
standard deviation	3.69	3.00	3.28	2.90
median	6.24	4.31	5.78	4.78
95th percentile	12.97	10.55	11.83	10.03
	**gts**	**gtr**	**gfs**	**gfr**
mean	5.69	4.74	5.49	4.90
standard deviation	3.20	2.89	3.06	2.90
median	5.29	4.57	5.19	4.69
95th percentile	12.23	9.69	11.31	10.46
**Entropy (bit)**	**nts**	**ntr**	**nfs**	**nfr**
mean	0.03	1.94	0.19	3.07
standard deviation	0.13	1.13	0.36	1.09
median	5.45 × 10^−30^	2.00	1.21 × 10^−30^	3.35
95th percentile	0.07	3.68	1.00	4.49
	**gts**	**gtr**	**gfs**	**gfr**
mean	0.63	3.17	0.68	3.25
standard deviation	0.64	1.23	0.67	1.05
median	0.46	3.41	0.54	3.44
95th percentile	1.77	4.76	1.92	4.59

**Table 5:** UJIIndoorLoc summarizing table. The column labels refer to using the normal sensor model (n) or using the convolution sensor model (g), using the trained *σ* (t) or using the fixed *σ* (f) and using a single reference location for training (s) or using a region of reference locations for training (r).

Error (m)	nts	ntr	nfs	nfr
mean	12.58	13.11	9.86	11.14
standard deviation	14.81	13.33	10.98	14.89
median	7.93	8.69	6.96	7.786
95th percentile	39.09	41.16	28.80	31.02
	**gts**	**gtr**	**gfs**	**gfr**
mean	10.49	12.93	9.19	10.52
standard deviation	11.54	12.95	10.58	13.82
median	6.85	8.84	6.22	7.67
95th percentile	32.10	40.29	26.10	30.99
**Entropy (bit)**	**nts**	**ntr**	**nfs**	**nfr**
mean	0.15	1.27	0.94	2.23
standard deviation	0.33	0.83	0.72	0.77
median	3.90 × 10^−6^	1.34	0.91	2.28
95th percentile	0.97	2.55	2.19	3.41
	**gts**	**gtr**	**gfs**	**gfr**
mean	0.24	1.23	0.97	2.08
standard deviation	0.45	0.90	0.72	0.74
median	1.26 × 10^−5^	1.28	0.96	2.13
95th percentile	1.32	2.65	2.24	3.13

**Table 6:** Quality measures of the sensor model implementations in the different environments. The column labels refer to using the normal sensor model (n) or using the convolution sensor model (g), using the trained *σ* (t) or using the fixed *σ* (f) and using a single reference location for training (s) or using a region of reference locations for training (r).

Environment	nts	ntr	nfs	nfr
EVARILOS	0.510	0.985	0.686	0.995
GEOTEC	0.002	0.622	0.026	0.859
UJIIndoorLoc	0.217	0.790	0.802	0.972
	**gts**	**gtr**	**gfs**	**gfr**
EVARILOS	0.711	0.959	0.995	0.995
GEOTEC	0.093	0.837	0.159	0.863
UJIIndoorLoc	0.280	0.742	0.810	0.970
